# The First Reported Case of Post-Atrioventricular Node Ablation Enterococcus Faecalis Bacteremia in a Patient With Colonic Tubular Adenomas and Chronic Steroid Use

**DOI:** 10.7759/cureus.20549

**Published:** 2021-12-20

**Authors:** Soban Ahmad, Madeleine Cutrone, Sundus Ikram, Sara Yousaf, Amman Yousaf

**Affiliations:** 1 Internal Medicine, East Carolina University/Vidant Medical Center, Greenville, USA; 2 Internal Medicine, East Carolina University Health Sciences, Greenville, USA; 3 Medicine, SEGi University, Petaling Jaya, MYS; 4 Anesthesiology and Critical Care, Kaul Associates, Lahore, PAK; 5 Internal Medicine, McLaren Flint, Flint, USA

**Keywords:** av node ablation, faecalis, steroid use, colonic adenoma, tubular, enterococcus

## Abstract

We present the case of a 73-year-old immunosuppressed male with a history of multiple benign, colonic adenomas who was admitted to our hospital with *Enterococcus faecalis (E. faecalis) *bacteremia*. *The patient also had a prior history of dual-chamber pacemaker placement for sick sinus syndrome. Two days before the admission, the patient had undergone radiofrequency ablation of the atrioventricular (AV) node for refractory atrial flutter without receiving any peri-procedural antibiotic prophylaxis. Despite high-grade bacteremia and a high NOVA (Number of positive blood cultures, Origin of the bacteremia, previous Valve disease, Auscultation of heart murmur) score, there was no evidence of infective endocarditis on transesophageal echocardiogram (TEE). The patient was treated successfully with appropriate intravenous antibiotics, and he recovered well.

To the best of our knowledge, this is the first reported case of post-AV node ablation *E. faecalis* bacteremia. We conclude that the presence of colonic lesions and immunosuppression can increase the risk of peri-procedural *E. faecalis* bacteremia, and clinicians should consider antibiotic prophylaxis in this high-risk patient group.

## Introduction

*Enterococcus faecalis *(*E. faecalis*), formerly classified as part of the group D *Streptococcus* system, is becoming a leading cause of bacteremia among older patients. *E. faecalis* is the most common species responsible for a majority (61-80%) of *Enterococci* infections [1]. Although colonic malignancy is the most frequent comorbidity in patients with *E. faecalis* infection, there have been few descriptions of *E. faecalis* bacteremia in patients with pre-existing colonic lesions [1,2]. There is scant evidence in the literature as to whether the presence of intestinal neoplasm and immunosuppression increases the risk of bacteremia in the setting of invasive cardiac procedures. According to our PubMed search, there has been no reported case of *E. faecalis* bacteremia following cardiac electrophysiologic procedures.

In this report, we describe a case of post-atrioventricular (AV) nodal ablation *E. faecalis* bacteremia in a patient with a history of colonic lesions and chronic steroid use. We also engage in a brief review of the pertinent literature.

## Case presentation

A 73-year-old male was urgently transferred to our hospital because of high-grade fever, rigors, and hypotension. Two days before the presentation, the patient had undergone radiofrequency ablation of the AV node through right femoral vein access for refractory atrial fibrillation. Appropriate sterile precautions had been taken during the procedure, including access-site cleansing with chlorhexidine solution. His past medical history was also significant for dual-chamber pacemaker implantation three years ago for sick sinus syndrome. In addition, he was taking chronic steroid therapy (prednisone 40 mg daily) for pulmonary fibrosis secondary to coronavirus disease 2019 (COVID-19) and long-term amiodarone use. Of note, the patient also had a history of multiple, low-grade, tubular adenomas removal on screening colonoscopies performed two and seven years ago. He denied any other urologic or gastrointestinal procedures.

**Figure 1:**
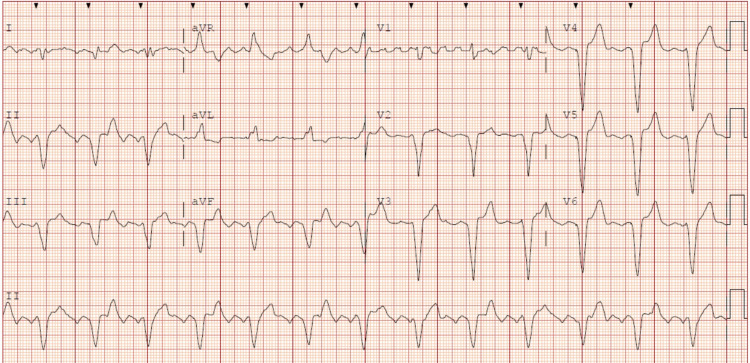
12-lead electrocardiogram showing atrial flutter waves with ventricular paced rhythm

On physical examination, his initial vital signs at the outside hospital had been as follows; blood pressure of 125/79 mmHg, heart rate of 77 beats per minute, respiratory rate of 18 breaths per minute, and temperature of 103.1 °F. His oxygen requirement was at baseline, 4 L per minute, with an oxygen saturation of 98%. Cardiac auscultation did not reveal any murmur. Lung auscultation revealed bilateral, diffuse fine crackles suggestive of underlying pulmonary fibrosis. Skin examination was unremarkable for erythema, tenderness, or purulent discharge at the right groin access site and the pacemaker site in the left anterior chest wall. On admission, laboratory testing revealed elevated white blood cell count (12.98 x 10^3^/µl), hemoglobin of 12.7 g/dl, elevated lactic acidosis (6.6 mmol/L), C-reactive protein (17.3 mg/L), and troponin I (0.64 ng/ml; normal: <0.03 ng/ml). His basal metabolic panel was unremarkable. Electrocardiography revealed atrial flutter with ventricular-paced rhythm (Figure 1).

Soon after the admission, the patient’s blood pressure dropped to 75/43 mmHg. Blood cultures and urine cultures were collected as part of the septic workup. He was treated with intravenous normal saline boluses, norepinephrine infusion, and was admitted to the cardiac ICU. He was also started on empiric antibiotics: linezolid (due to a known history of vancomycin allergy) and piperacillin-tazobactam. The patient responded well to the above treatment. However, his troponin I level peaked at 0.98 ng/ml, likely from an underlying demand-supply mismatch. Within 16 hours of admission, his blood cultures grew gram-positive cocci that were later identified as *E. faecalis*. No bacterial growth was noted in urine culture. The antibiotic regimen was de-escalated to daptomycin. A transthoracic echocardiogram (TTE) did not reveal any new valvular abnormalities or vegetations.

**Figure 2:**
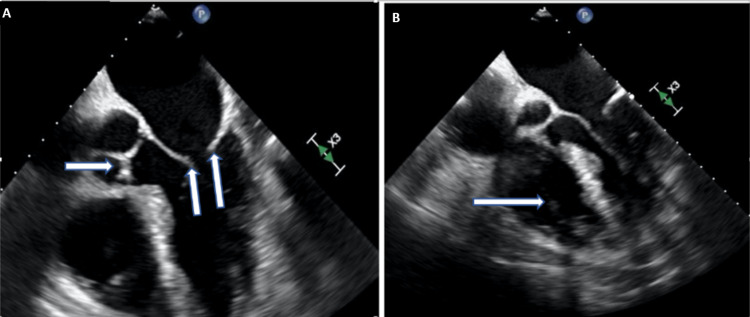
Transesophageal echocardiogram (four-chamber views) 2A. This view shows no evidence of vegetations involving mitral valve leaflets (vertical arrows) or aortic valve cusps (horizontal arrow). 2B. This view displays no obvious vegetations over the right ventricular pacemaker lead (arrow)

Repeat blood cultures post-48 hours of antibiotic therapy remained positive. At this point, our hospital’s infectious disease team was consulted, who recommended a transesophageal echocardiogram (TEE) to rule out infective endocarditis due to a calculated NOVA (Number of positive blood cultures, Origin of the bacteremia, previous Valve disease, Auscultation of heart murmur) score of 9. No evidence of valvular or pacemaker lead vegetations was seen on TEE (Figure 2).

Blood cultures were repeated every 24 hours until the clearance of bacteremia was achieved on the sixth day of hospitalization. Antibiotic therapy was further de-escalated to IV penicillin G 24 million units per day based on the sensitivity report and a favorable minimum inhibitory concentration level (MIC: 2 µg/ml). The patient was discharged home on day seven of admission in a stable condition. He has completed two weeks of intravenous antibiotic therapy and continues to do well to date.

## Discussion

*Enterococci* are gram-positive commensals of the gut flora and a frequent cause of bacteremia in the nosocomial setting, causing about 12% of healthcare-associated bloodstream infections [3]. *Enterococcal* species are the second most common cause of surgical site infections after *Staphylococcus aureus*, but this risk is minimal with proper sterile techniques. *Enterococcal* bacteremia can also occur endogenously due to translocation from normal gut flora through the intestinal mucosa to the lymphatics and bloodstream or via direct inoculation during medical procedures without proper sterilization [3].

Patients with *enterococcal* bacteremia are at significantly increased risk of developing infectious endocarditis (IE), with a recent study reporting a 26% prevalence in these patients [4]. Considering the high incidence of endocarditis with *Enterococcus* bacteremia, many patients require further evaluation with TEE. The likelihood of IE and thus the need for TEE can be determined using the NOVA score [5]. Our patient had a NOVA score that warranted further evaluation with TEE but was not found to have any evidence of endocarditis on echocardiography.

Our patient had a known history of colonic tubular adenomas discovered on colonoscopy. Although a causal link has not been established between *enterococcal* bloodstream infections and colonic dysplasia, there are several case reports and retrospective studies indicating a relationship between the two [2,6-8]. Although currently unclear, *enterococcal* proliferation in the setting of colonic lesions may allow for increased translocation of gut flora into the bloodstream. Alternatively, there is also data demonstrating carcinogenic properties of e*nterococci* that induce cellular changes leading to dysplasia in colonic tissue [9]. Our patient, therefore, had multiple independent risk factors for *enterococcal* bacteremia, including an immunocompromised state, colonic adenomas, and a recent medical procedure.

Bloodstream infection after cardiac catheterization procedures, including AV node ablation, is very uncommon if proper sterile techniques are used, which had been ensured in our patient. For electrophysiologic studies specifically, the incidence is only about 0.8% [10]. Risk factors for *enterococcal* bacteremia post-AV node ablation that apply to our patient include the presence of colonic adenomas and immunosuppression. Prophylactic antibiotics are routinely recommended before and after implanting cardiac electronic devices such as pacemakers but not for other catheterized procedures, including ablations [11]. Based on current guidelines, prophylactic antibiotics had not been used in our patient, and he developed bacteremia following AV node ablation.

## Conclusions

We conclude that patients with a history of colonic adenomas and chronic corticosteroid use might be at increased risk for *enterococcal* bacteremia from the translocation of gut flora following an invasive cardiac procedure. We suggest that clinicians should consider the use of prophylactic antibiotics in patients with colonic adenomas and immunosuppressed status before performing elective cardiac catheterization procedures. Also, given the inconclusive data in the literature so far, further studies should be performed to explore the actual incidence, pathophysiology, and prevention of this association.
